# Sensory Analysis of Vegan Acibadem Cookie Produced From Aquafaba in Terms of Consumers and Evaluation of Purchase Intention

**DOI:** 10.1002/fsn3.70141

**Published:** 2025-04-21

**Authors:** Samet Can Aksu, Yılmaz Seçim

**Affiliations:** ^1^ Department of Gastronomy and Culinary Arts Tourism Faculty, Ordu University Ordu Türkiye; ^2^ Department of Gastronomy and Culinary Arts, Tourism Faculty Necmettin Erbakan University Konya Türkiye

**Keywords:** aquafaba, purchase intention, vegan, willingness to pay premium price

## Abstract

In this study, a standard recipe for the vegan version of acibadem cookies, one of the important desserts in Turkish cuisine, was created. Within the scope of this study, the impact of sensory attributes on purchase intention was examined through the willingness to pay premium price. While investigating the effects of sensory attributes on purchase intention, it was also explored whether demographic factors such as income perception and dietary style revealed significant differences. The vegan product developed as part of the research contains no animal products and includes chickpea cooking water, known as aquafaba. Thus, the product was developed in accordance with the zero‐waste principle and made suitable for all dietary styles. The primary aim here was to develop an environmentally friendly product that also provides sensory satisfaction suitable for all dietary preferences. For this purpose, sensory analysis was conducted with 40 vegan, 40 vegetarian, and 40 omnivore participants. According to the research data, the overall appreciation for the developed product was rated as “good,” with scores of 4 and above across all dietary styles. Vegans showed the highest purchase intention among the dietary groups, followed by vegetarians. Regression analysis showed that the overall taste of the product significantly influenced purchase intention. It was found that if the sensory attributes of a developed vegan food product are liked, there is a willingness to pay a premium price across all dietary styles. The results show that if a product with a certain sensory competence is produced, regardless of the dietary style it caters to, it can achieve a high purchase intention. This outcome is thought to provide insights for companies producing vegan products on how they can appeal to a broader market.

## Graphical abstract

This study aims to create a product that appeals to all dietary styles using vegan substitute ingredients such as aquafaba. The research demonstrates that it is possible to develop a product that can cater to all dietary preferences, even when using vegan ingredients, provided that the necessary sensory attributes are met.

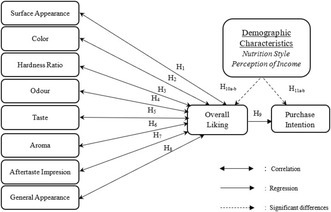

## 1 Introduction

The vegetarian diet is gaining popularity, especially in Western countries (Al‐Mohaithef 2022). According to 2022 data, approximately 5% of the US population (15.5 million) follow a vegetarian diet, while 2 million people in this group have a vegan diet (Statista 2023). The pandemic, which has affected everyone globally, has also been found to have contributed to the increase in the vegetarian and vegan population by making some people more aware of healthier diets (Loh et al. 2021). According to a nutrition report published in Germany, the number of vegetarians and vegans doubled after COVID‐19 (Wirnitzer et al. 2022). In parallel to this situation, the vegan product market shows a growth trend every year. This trend has encouraged leading food manufacturers in the food market to develop innovative vegan food products. The ongoing transition also reinforces the sustainability transformation of the food sector (Saari et al. 2021). Other reasons for the expansion of the vegan product market include increased awareness of animal rights and living standards, greater attention to personal health, and increased awareness of environmental damage (Ploll and Stern 2020). Growing concerns affect not only vegans but also nonvegan consumers. Research shows that nonvegan people are also more willing to pay premium prices for vegan products (Martinelli & De Canio, 2021). The fact that food and beverage establishments in the sector have started to include vegan/vegetarian products in their menus regardless of their style and concept proves that the demand is increasing in this direction (Ginsberg 2017). When food and beverage types were analyzed in terms of sustainability, it was found that the environmental impact of a vegetarian menu was approximately 2.8 times lower than an omnivorous menu. At the same time, the impact of a vegan menu was found to be 3.3 times lower than that of an omnivorous menu (Ruini et al. 2015). It is stated that including vegan products in the menus of food and beverage establishments will contribute to environmental sustainability (Niederle and Schubert 2020).

In line with this information, the vegan acibadem cookie was developed as an alternative product for the growing vegan product market. In the developed cookie, chickpea juice is used instead of egg whites. Thus, both the carbon footprint of egg production is reduced and the water from chickpeas is utilized, contributing to sustainability as a result of the zero‐waste practice. The sensory analysis of the product developed for this purpose was carried out, and the general level of appreciation was measured. At the same time, in order to contribute to sustainability, it was aimed to develop a product that not only vegans but people of all dietary styles would like and want to buy. For this purpose, in addition to sensory analysis, the effect of overall liking on purchase intention was measured, and differences between dietary styles were analyzed.

## 2 Theoretical Background

### 2.1 Veganism and Vegan Product Market

Veganism is defined as a philosophy based on the nonconsumption of animal foods. This philosophy is grounded in the idea that animals are not objects and that animal consumption harms the environment (Vestergren and Uysal 2022). Veganism, a subtype of vegetarianism, has been practiced for various ethical, environmental, health, philosophical, and spiritual reasons throughout history and has become a widespread phenomenon not only in Western societies but also in many cultures around the world (Gheihman 2021). Environmental concerns are driven by the fact that all activities from food production to consumption contribute to around 30% of human‐induced climate emissions, exacerbating environmental problems such as freshwater withdrawals, nutrient pollution, and biodiversity degradation (Clark et al. 2019). All these factors are raising the demand for vegan products and driving the growth of the vegan market (Martinelli and de Canio 2022). As a result of the increasing demand for vegan products, companies are diversifying their production and developing diverse alternatives (Saari et al. 2021). In addition to brands such as Beyond Meat, where a completely vegan initiative is at the forefront, Nestlé, one of the major food companies, has also started producing vegan products alongside its regular products (Kumari and Muralidhara 2021; Mudry and Phillips 2023). Particularly after the COVID‐19 pandemic, people's diets have changed and they have sought healthier ways of eating (Eftimov et al. 2020). At this point, the increase in demand for plant‐based products has positively affected the development of the vegan product market (Noguerol et al. 2021).

### 2.2 Aquafaba

Inadequate access to products with protein content is recognized as one of the most significant nutritional problems in the world. Legumes are valuable sources of protein in terms of content. Recycling legume cooking water as raw material is an alternative way to make more sustainable use of limited resources (Erem et al. 2023). Aquafaba has become popular among vegans, especially due to its use as an egg white substitute, but it also has nutritious content (Raikos et al. 2020; Rondoni et al. 2021). The term aquafaba was first used in the scientific literature by Shim et al. (2018). Aquafaba, the liquid obtained by cooking chickpeas in water, is usually discarded. However, since aquafaba is a valuable ingredient with functional properties such as emulsifying, foaming, and gelling, it is used in puddings, mayonnaise, ice cream, dairy desserts, and bakery products as an alternative to eggs and milk in vegan products (Mustafa et al. 2018).

### 2.3 Willingness to Pay Premium Price and Purchase Intention

Willingness to pay a premium price (WTP) is defined as the readiness to pay more to obtain a particular product or service among comparable alternative brands (Dwivedi et al. 2018). WTP is also one of the most important determinants of purchase behavior and intention (Netemeyer et al. 2004). Consumers are willing to purchase the same product or brand in the future if they are satisfied with the service or product they have purchased or if their expectations for the product are met (Fornell et al. 2010). Consumers are particularly willing to pay more for products related to important issues that affect society as a whole, such as environmental concerns and human health. For some consumers, their favorite brands are unique. Therefore, given market conditions, they are willing to pay more for their favorite product, even if brands selling a comparable product in the market have more appealing benefits (Siew et al. 2018). Other factors influencing the willingness to pay more include the level of quality of the goods or services, the level of consumer trust, and food safety (Mulder and Zomer 2017; Joya et al. 2022).

## 3 Research Model and Hypotheses

### 3.1 Sensory Characteristics of the Product and Overall Liking

The concept of overall liking is scientifically interpreted as a holistic hedonic response in which the consumer evaluates the attractiveness of the product. A limited number of studies have been conducted to understand whether liking all four general sensory attributes (appearance, odor, taste, and texture) is reflected in the overall evaluation of liking or whether a single sensory attribute stands out (Andersen et al. 2019).

Conti‐Silva and de Souza‐Borges (2018) examined the effect of sensory characteristics of four commercial probiotic fermented milk on the overall taste of the product and concluded that the sensory profile affects the overall taste of probiotic fermented milk. Nguyen and Wismer (2019) compared the sensory profiles and overall liking between regular and reduced sodium potato chips, pickles, cooked ham, and chicken noodle soup products. While it was concluded that sodium‐reduced pickles were liked more than regular pickles, 25.5% of the participants rated the sodium‐reduced ham as not salty enough, and this low level of salinity reduced the overall liking of some participants. Ribeiro et al. (2019) used different drying methods and insect species to improve the sensory characteristics of insect‐containing cereal bars. Bars containing defatted or microwaved mealworms had a similar willingness to eat scores and liking compared to the control bar, while bars containing oven‐dried insects were rated worse. The use of defatted insects in the cereal bar and microwave oven drying eliminated negative flavor attributes by preventing undesirable odors. Therefore, the following hypotheses are formulated:

*There is a significant relationship between the surface appearance sensory characteristic of vegan acibadem cookie and the overall liking of the cookie*.

*There is a significant relationship between the color sensory characteristic of vegan acibadem cookie and the overall liking of the cookie*.

*There is a significant relationship between the sensory characteristic of hardness ratio of vegan acibadem cookie and the overall liking of the cookie*.

*There is a significant relationship between the odor sensory characteristic of vegan acibadem cookie and the overall liking of the cookie*.

*There is a significant relationship between the taste sensory characteristics of vegan acibadem cookie and the overall liking of the cookie*.

*There is a significant relationship between the aroma sensory characteristics of vegan acibadem cookie and the overall liking of the cookie*.

*There is a significant relationship between the aftertaste impression sensory characteristics of vegan acibadem cookie and the overall liking of the cookie*.

*There is a significant relationship between the general appearance sensory characteristic of vegan acibadem cookie and the overall liking of the cookie*.

### 3.2 The Effect of Nutrition Style and Perception of Income on Overall Liking

Consumers' food choices are economically driven decisions shaped by taste, income, reward value, price, and perceived nutritional value of foods (Drewnowski and Monsivais 2020). Dovi et al. (2018) developed sorghum–cowpea composite biscuits for people with malnutrition and compared the sensory profiles of these biscuits with economical commercial refined wheat biscuits through hedonic ratings of low‐income consumers. As a result of the research, it was found that consumers liked wheat biscuits more. Ma et al. (2018) also investigated the correlation between income and spicy food preference. They concluded that low‐income individuals preferred spicier foods compared to high‐income individuals, despite being in the same geography.

Another important factor in product preference is sensory decisions. These decisions may vary with dietary style as well as demographic characteristics. Especially in vegan and vegetarian individuals, the reaction to salty and sweet flavors is more pronounced (Leshem and Shaul 2022). Jalil Mozhdehi et al. (2021) measured taste perception thresholds for six compounds (sweet–sucrose, sour–citric acid, salty–sodium chloride, umami–monosodium glutamate, bitter–caffeine, MSG and metallic–iron II sulfate heptahydrate) with a total of 80 healthy New Zealand European women classified as 22 vegans, 23 vegetarians, and 35 omnivores. The study found that the omnivore group had different patterns of taste sensitivity across the six compounds compared to the vegetarian or vegan group. Furthermore, the vegetarian group had a considerably lower detection threshold for bitterness (i.e., caffeine) than the other two groups. Based on this discussion, the hypotheses to be tested in this study are as follows:

*There is a significant difference between the participants' overall liking and nutrition styles*.

*There is a significant difference between the participants' overall liking and perception of income*.

### 3.3 The Effect of Nutrition Style and Perception of Income on Purchase Intention

Sociodemographic factors such as lifestyle, occupation, age, and education have a significant impact on purchase intention and purchase behavior (Singh and Verma 2017; Slabá 2019). Individuals' income level also affects their purchasing behavior (Chowdhury et al. 2021). Low income constrains access to sufficient quantities of high‐quality food by restricting a flexible portion of the household budget (Śmiglak‐Krajewska et al. 2020). Low‐income households purchase less healthy foods than high‐income households, which may mediate income differences in food purchasing patterns and dietary intake quality (French et al. 2019).

Yilmaz‐Ersan et al. (2020), in order to better understand consumers' attitudes, investigated consumers' knowledge and awareness of probiotic dairy products, considering their sociodemographic characteristics and health status. It was observed that those with higher education and income levels had higher awareness and knowledge of probiotic dairy products, which increased the likelihood of purchasing probiotic dairy products. Bryła (2018) analyzed selected characteristics, attitudes, and opinions of organic food e‐consumers (online shoppers) in Poland. They found that higher income and higher WTP increase the possibility of becoming an organic e‐consumer. As a result of the findings in the literature, the hypotheses to be tested in this study are as follows:

*There is a significant difference between the participants' purchase intentions and nutrition styles*.

*There is a significant difference between the participants' purchase intentions and perception of income*.

### 3.4 Overall Liking and Purchase Intention

The overall liking variable is used to measure consumers' overall hedonic response to food (Andersen et al. 2019). Overall liking positively influences willingness to pay more, purchase intention, and acceptability (Grasso et al. 2022).

Montouto‐Graña et al. (2012) aimed to determine purchase intention by obtaining consumers' opinions about fresh‐cut potatoes packed under a vacuum and under a modified atmosphere, taking into account the sensory characteristics of the potato. The results show that although both products are well accepted by consumers, vacuum‐packed potatoes score relatively higher and more consumers intend to purchase this product. Bezerra et al. (2017) aimed to evaluate the effects of thermal and nonthermal treatments applied to Açai fruits on physicochemical traits, sensory quality, and acceptance of processed Açai beverages. As a result of the study, consumers were incapable of distinguishing between thermally processed and chlorination‐sterilized beverages. At both temperatures, the beverage from blanched fruits obtained good ratings and positive purchase intention. Anetoh et al. (2020) examined the influence of tasting, visual, tactile, and olfactory attributes of malt brands on consumer purchase intentions in Nigeria. The findings indicated that the visual, gustatory, tactile, and olfactory properties of malt brands have significant impacts on consumers' purchase intentions. Based on this argument, the following hypothesis is proposed:

*There is a significant relationship between the participants' overall liking for acibadem cookie and their purchase intention*.

## 4 Materials and Methods

### 4.1 The Design of the Survey and Data Collection

Within the scope of the study, four different forms of acibadem cookies that can be consumed by vegans were prepared with almond flour, hazelnut flour, powdered sugar, and chickpea juice (aquafaba) used as an egg white substitute. The evaluation part takes place in two stages. First, a group of (eight) trained panelists consisting of Ordu University Department of Gastronomy and Culinary Arts faculty members and graduate students tasted four different vegan acibadem cookies with codes 101, 201, 301, and 401. As a result, sensory analysis of these products was carried out, and the most liked product was determined. In the second stage, the most liked form was turned into a standard recipe, and sensory analysis was made by consumers (120) to evaluate the liking of a new product and measure their purchase intentions. The questionnaire consists of three parts. In the first part, questions to determine the demographic characteristics of the participants were included. In the second part, the product surface appearance, color, hardness, smell, taste, flavor, aroma, the impression left in the mouth after tasting, general appearance, and overall liking level were asked in the form of a 5‐point liking scale. In the third section, questions were asked to measure purchase intentions about the tasted product. (Table 1).

**TABLE 1:** Respondent profile.

Demographic characteristics		*N*	%
Gender	Female Male	70 50	58,3 41,7
Age	18–25 26–45 46–64 65+	56 54 7 3	46,7 45,0 5,8 2,5
Education	Elementary High school Associate degree Bachelor's degree Master's degree PhD	1 4 25 81 5 4	0,8 3,3 20,8 67,5 4,2 3,3
Income perception	Low Medium High	39 27 54	32,5 22,5 45,0
Marital status	Married Single	32 88	26,7 73,3
Nutrition style	Vegan Vegetarian Omnivore	40 40 40	33,3 33,3 33,3

### 4.2 Sample Selection

In this study, the appropriate number of panelists (sample size) was selected based on the specific requirements of the study and the expertise needed for the evaluation. In the first part of the study, 4 different cookies prepared for vegan consumers were tested by 8 trained panelists. Panelists consisted of people who were well‐informed in sensory analysis, working as faculty members in the department of gastronomy and culinary arts or having a master's degree. Convenience sampling and criterion sampling methods were used to determine the sample group of 120 people in the second part of the study. The convenience sampling method is one of the nonrandom sampling methods. The sample group is determined by the researcher (Malhotra and Dash 2016). The understanding of the criterion sampling method is that all situations that meet predetermined criteria are studied. The reason for choosing the “criterion sampling” method in the study is that the data can be obtained from people with vegan and vegetarian diets in line with the purpose of the research (Patton 2005). While the convenience sampling method was used to reach (40) consumers who do not adopt a vegan or vegetarian diet, the criterion sampling method was used to reach only (40) consumers who follow a vegan (40) or vegetarian (40) diet.

### 4.3 Measures

In the sensory analysis form prepared by examining the thesis of Seferoğlu (2012), sensory quality criteria (5 points: very good, 4 points: good, 3 points: neither good nor bad, 2 points: bad, and 1 point: very bad) were measured using a 1–5 scoring test. Willingness to pay more is one of the most important determinants of purchase behavior and purchase intention in consumers (Netemeyer et al. 2004). From this point of view, consumers' purchase intentions were attempted to be measured by their willingness to pay more. The willingness to pay more for vegan products scale, adapted by Martinelli and de Canio (2022), was used to measure purchase intention for vegan acibadem cookies. Participants were asked to respond to the four statements on a 1–5 hedonic scale (5 points: strongly agree, 4 points: agree, 3 points: undecided, 2 points: disagree, and 1 point: strongly disagree).

### 4.4 Raw Materials

For the developed product, four different cookies were first produced from two different flours. The main purpose of creating four different cookies in the first stage was to make vegan acibadem cookies with both almond flour and hazelnut flour. By using hazelnut and almond flour, experimental, not proportional, differentiation was made in the material content of the cookies. Thus, it was aimed to provide different flavors and product textures, to offer variety to tasters, and to reach a good product. Chickpea (aquafaba) juice was used instead of the egg white in the standard acibadem cookie to obtain a product close to the acibadem cookie.

Within the scope of the research, cookies were coded as 101, 201, 301, and 401. The visuals, contents, product codes of the cookies produced, and the general appreciation results of the products by experienced panelists are given in Table 2.

**TABLE 2:** Result of first sensory analysis stage.

Ingredients	Product code	Overall liking rate
130 g almond flour		
35 mL chickpea water	101	3.5
40 g powdered sugar		
130 g hazelnut flour		
35 mL chickpea water	**201**	**4.5**
40 g powdered sugar		
130 g almond flour		
55 mL chickpea water	301	4.25
30 g powdered sugar		
130 g hazelnut flour		
55 mL chickpea water	401	3.825
60 g powdered sugar		

*Note:* Bold value indicates highest overall liking score.

The cookies, prepared with various ingredients, were presented to the panelists and subsequently ranked according to the results of the sensory analysis conducted by the panelists. The product made with hazelnut flour, coded 201, was the most preferred, while the product made with almond flour, coded 101, was the least preferred. Based on its average rating of 4.5, it was decided to further develop the product coded 201 and to assess consumers' purchase intention. In line with the data, it was decided to develop the product coded 201 and to measure the purchase intention of the product.

#### 4.4.1 Cookie Production

The powdered sugar should be added gradually to the chickpea water and mixed until a thick consistency is achieved. The flour should be added to the aquafaba in two portions and gently folded from the outside to the inside. The dough should then be portioned into walnut‐sized pieces and placed on the baking tray. It should be baked at 170° for 20 min.

#### 4.4.2 Sensory Analysis Process

In the first part of the study, four different cookies were produced using two different types of flour. In order to eliminate all factors that could affect the sensory decisions of the trained panelists, the cookies were tested in a sensory tasting room. The products were presented to the panelists in a random order, and each cookie was served in a 30‐g portion. During the tasting session, water and bread were provided to help cleanse the panelists' palates. The panelists assessed the products based on appearance, aroma, texture, taste, and overall liking. The products were evaluated using a 1–5 rating scale. The cookie with the highest score for overall liking was included in the second stage of the consumer taste survey. At this stage, to eliminate all factors that could influence consumers' sensory decisions, the sensory analysis was conducted in the sensory tasting room, as it was in the first stage. Since consumers were only tasting a single product, no neutralizing products were provided. A group of eight trained panelists, who were experienced in sensory evaluation, assessed the product solely based on its sensory properties, while the consumer survey also measured the effect of overall liking on purchase intention.

### 4.5 Statistical Analysis

**FIGURE 1:**
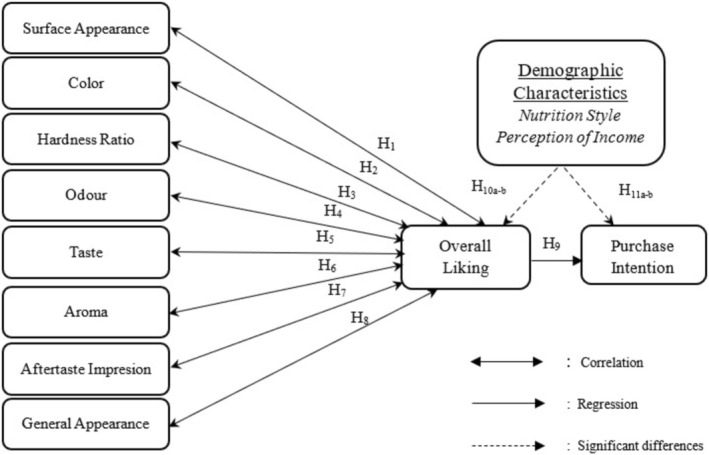
Research model.

The analysis of the data obtained within the scope of the research was evaluated in the SPSS 23.0 (Statistical Package for Social Sciences) program. Descriptive statistical methods of the program (frequency, percentage, mean, and standard deviation) were used to evaluate the data. In order to investigate the effect of the sensory characteristics (surface appearance, color, hardness, odor, taste, aroma, aftertaste impression, general appearance, and overall liking) of vegan acibadem cookies produced within the scope of the study on purchase intention, the data were evaluated using SPSS 23.0 statistical program. In the analysis part of the data obtained, “Pearson Correlation Analysis” was used to examine the relationship between sensory characteristics and purchase. When comparing the data obtained, t‐test between groups was used when there were only two dependent variables. In cases where the variables consist of at least three or more groups, analysis of variance (ANOVA), which is the analysis of differences between groups, was used. In addition, Tukey HSD test was used to determine between which groups the difference was between. “Linear Regression Analysis” was used to measure the effect of general liking on purchase intention. Regression analysis demonstrates the extent to which a dependent variable is affected by one or more independent variables. Regression analysis is also used to determine how much a change in the dependent variable explains a change in the independent variable and to determine how much of the change in the dependent variable is caused by the dependent variable. (Allison 1990). Within the scope of this study, since the effect of the independent variable, overall liking, on the dependent variable, purchase intention, was to be measured, the use of regression analysis was deemed appropriate. (Figure 1).

## 5 Data Analysis and Results

### 5.1 Sensory Analysis

In line with the answers given by the participants included in the sensory analysis, the results of the sensory analysis for the vegan acibadem cookie are given in Table 3. Participants scored the cookie as very bad–very good/1–5) except for the hardness ratio. The hardness ratio is rated as very hard–very soft/1–5.

**TABLE 3:** Sensory analysis results.

Sensory properties	Vegan	Vegetarian	Omnivore
Odor	4,5	4,275	4,525
Aroma	4,625	4,6	4,475
Color	4,375	4,3	4,45
Taste	4,65	4,6	4,475
General appearance	4325	4,475	4,3
Hardness ratio	3,575	3,475	3,175
Aftertaste	4,55	4,475	4,35
Overall liking	4,6	4625	4,45

**FIGURE 2:**
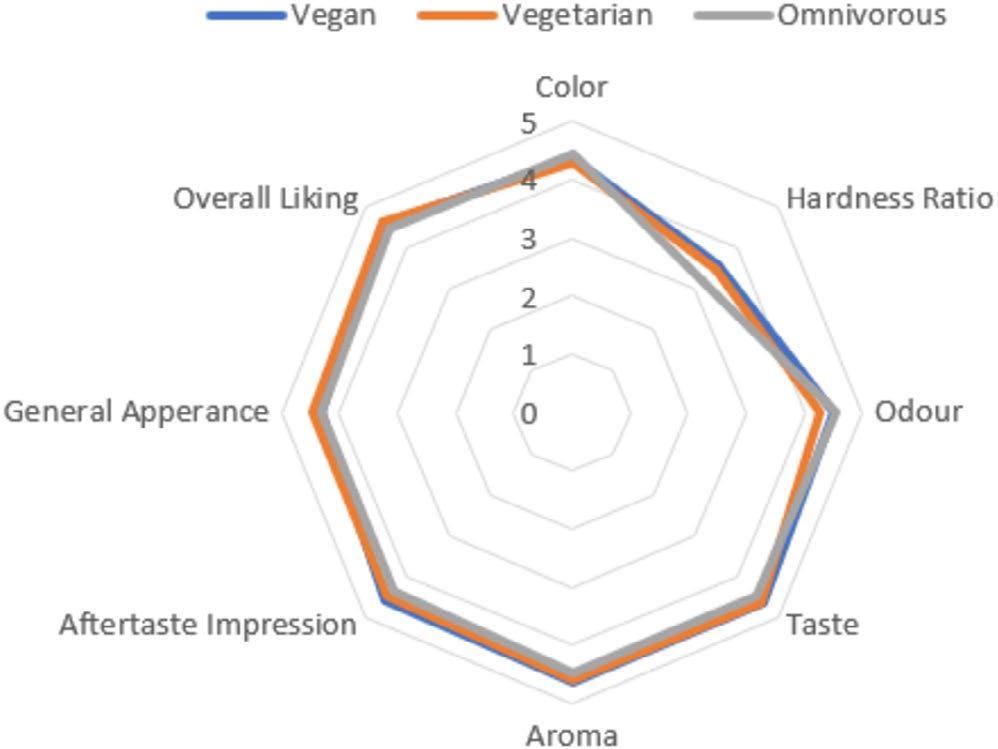
The spider web graph of sensory analysis results.

Considering the overall average of the answers given by 120 participants in the sensory analysis phase, the hardness of the vegan acibadem cookie was 3 and above, and the sensory characteristic was described as “neither hard nor soft.” The other sensory attributes (color, general appearance, smell, aroma, odor, flavor, and aftertaste impression) scored 4 and above and were described as “good.” Considering the overall taste of all participants, the developed vegan acibadem cookie was found to be “good” with a score of 4 and above. (Figure 2).

When the sensory analysis results are analyzed, it is observed that although there is no significant difference between dietary styles, all participants gave consistent answers in terms of the sensory properties of the product. In the evaluation conducted on a 5‐point scale, regarding the aroma characteristic, vegans scored it 4.625, vegetarians gave it a 4.6, and omnivores assigned it 4.475. For color, vegans noted it as 4.375, vegetarians assessed it at 4.3, and omnivores marked it at 4.45. As for flavor, vegans evaluated it at 4.65, vegetarians provided a score of 4.6, and omnivores indicated a rating of 4.475. Regarding the odor characteristic, vegans scored it 4.5, vegetarians assessed it at 4.275, and omnivores gave it a 4.525. In terms of hardness ratio, vegans rated it at 3.575, vegetarians assessed it at 3.475, and omnivores scored it at 3.175. The product is perceived as softer by omnivores than by those following other diets.

### 5.2 Analysis of Participants' Purchase Intentions for Vegan Acibadem Cookies

Reliability refers to the ability of a test or scale to consistently and stably measure the intended construct (Peter 1979, 6). In this context, Cronbach's alpha analysis is the most widely used method to measure the internal consistency of the statements that make up the measurement tool. The lower limit for the Alpha value to be within the acceptable range is accepted as 0.7 (Hajjar 2018). Factor analysis was applied to the statements in order to determine the purchase intention of the product tasted by the participants. The results of the factor analysis are presented in Table 4. Before conducting the factor analysis, some preliminary statistical tests were performed. The results of these tests showed that the KMO (Kaiser–Meyer–Olkin) value of the scale was 0.733, Bartlett's sphericity test yielded a value of 6 (df), and Cronbach's alpha value was 0.771. The results of these preliminary tests indicate that the scale is suitable for factor analysis and its construct validity is ensured.

**TABLE 4:** Reliability and validity scores of constructs (*n* = 120).

Variable		Component
Willingness to pay premium price (WTP)		
1	I am willing to pay more for vegan products even when a cheaper but not vegan alternative is available.	0.837
2	I make every effort to purchase this vegan product.	0.756
3	No matter its cost, I will buy this vegan product.	0.753
4	I am willing to buy this product in the future.	0.741
	Total variation explained Kaiser–Meyer–Olkin Cronbach's alpha Ave Barlett's test of sphericity	59.704 0.733 0.771 0.59 6

Correlation analysis was used to determine the relationship between the obtained sensory analysis dimensions and overall liking. Simple regression analysis was conducted to determine the level of influence of general liking on willingness to pay more.

The kurtosis and skewness values were calculated to examine the conformity of the scale scores to normal distribution. The obtained data are presented in Table 5.

**TABLE 5:** Test of normality.

	*n*	Min.	Max.	Mean	SD	Skewness	Kurtosis
WTP1	120	1	5	3,48	1123	−0.610	−0.203
WTP2	120	1	5	3,11	1067	−0.093	−0.782
WTP3	120	1	5	3,04	0.982	0.024	−0.633
WTP4	120	1	5	4,05	0.868	−1351	2357

When the kurtosis and skewness values were examined, the kurtosis and skewness values obtained from the scale scores were between +3 and −3, indicating that a normal distribution was realized (de Carlo 1997). In this regard, it was concluded that the values did not deviate excessively from the normal distribution, and the analyses were carried out with parametric tests.

Correlation analysis was applied between the variables. In the correlation analysis between the subdimensions related to sensory analysis: surface appearance, color, hardness ratio, odor, taste, aroma, aftertaste impression, general appearance, and overall liking variable, the significance level of the relationship between the data was evaluated as *p* = 0.01. As a result of the analysis, it was concluded that there was no significant relationship between overall liking and surface appearance (*r* = −0.137, *p* = 0.137) and hardness ratio (*r* = −0.154, *p* = 0.093). However, a positive and significant relationship was found between overall liking and the following variables: color (*r* = 0.420, *p* < 0.01), odor (*r* = 0.455, *p* < 0.01), taste (*r* = 0.494, *p* < 0.01), aroma (*r* = 0.700, *p* < 0.01), aftertaste impression (*r* = 0.604, *p* < 0.01), and general appearance (*r* = 0.439, *p* < 0.01). As a result of these data, while *H1* and *H3* were not accepted, *H2, H4, H5, H6, H7*, and *H*_*8*_ were accepted. (Table 6).

**TABLE 6:** Discriminant validity.

No	Constructs		1	2	3	4	5	6	7	8	9
1	Surface appearance	Pearson correlation	1								
		sig. (2‐tailed)	—								
2	Color	Pearson correlation	−0.028	1							
		sig. (2‐tailed)	0.760	—							
3	Hardness Ratio	Pearson correlation	−0.154	0.025	1						
		sig. (2‐tailed)	093	0.783	—						
4	Odor	Pearson correlation	−0.102	0.310^a^	−0.004	1					
		sig. (2‐tailed)	0.270	0.001	0.968	—					
5	Taste	Pearson correlation	−0.078	0.455^a^	−0.058	0.494^a^	1				
		sig. (2‐tailed)	0.399	0.000	0.530	0.000	—				
6	Aroma	Pearson correlation	−0.015	0.383^a^	−0.046	0.417^a^	0.700^a^	1			
		sig. (2‐tailed)	0.871	0.000	0.622	0.000	0.000	—			
7	Aftertaste impression	Pearson correlation	0.083	0.321^a^	0.087	0.433^a^	0.604^a^	0.589^a^	1		
		sig. (2‐tailed)	0.368	0.000	0.345	0.000	0.000	0.000	—		
8	General appearance	Pearson correlation	−0.071	0.439^a^	−0.046	0.272^a^	0.460^a^	0.410^a^	0.406^a^	1	
		sig. (2‐tailed)	0.443	0.000	0.621	0.003	0.000	0.000	0.000	—	
9	Overall liking	Pearson correlation	−0.137	0.420^a^	−0.024	0.289^a^	0.689^a^	0.545^a^	0.573^a^	0.541^a^	1
		sig. (2‐tailed)	0.137	0.000	0.794	0.001	0.000	0.000	0.000	0.000	—

^a^ Significant at the 0.01 level (two‐tailed).

In order to test the relationship between overall liking and willingness to pay more and the level of impact, simple regression analysis was applied. The findings of the simple regression analysis are presented in Table 7. As a result of the analysis, it is concluded that the ratio of the dependent variable (“overall liking”) and the independent variable (“willingness to pay premium price”) is sufficient (adjusted *R* ^2^ = 0.022). In addition, the Durbin–Watson coefficient (1.637) indicates that there is no autocorrelation problem. Also, when the other data were analyzed, it was concluded that *F* = 3653 and *p* = 0,000, and in this context, the model as a whole was significant at all levels. The relationship between variables is significant at 90% confidence interval. As a result of the analysis, *H*_*9*_ is accepted.

**TABLE 7:** Regression analysis of general liking on willingness to pay premium price.

	Beta	*t*‐value	*p*	Standard deviation	Tolerance value	VIF
Constant	2323	4014	0.000	0.579	—	—
Overall liking	0.241	1911	0,58	0.126	1000	1000
*R* ^2^	0,030					
Adjusted R^2^	0,022					
F	3653					
*P*	0.000					
Durbin Watson	1637					

ANOVA test was applied to determine whether there was a difference between the groups in the answers given according to the income perception of the participants, and the distributions of the results are given in Table 8. As a result of the analysis, a significant difference was found between the groups in terms of income perception in overall liking sensory characteristics. On the other hand, there was no significant difference between the groups in terms of willingness to pay premium price (*p* > 0.05). As a consequence of the results, *H*_*10b*_ is accepted while *H*_*11b*_ is not accepted.

**TABLE 8:** ANOVA results according to participants' income perception differences.

Subdimension		Sum of squares	df	Mean square	*F*	Sig.
Overall liking						
	Between groups Within groups Total	2361 35,231 37,592	2 117 119	1180 0.301	3920	0.022*
Willingness to pay premium price						
	Between groups Within groups Total	,240 72,383 72,623	2 117 119	0.120 0.619	0.194	0.824

*Note: p* < 0.05*.

ANOVA test was performed to determine whether there was a difference between the groups in the answers given according to the nutrition style of the participants, and the distributions of the results are given in Table 9. A significant difference was found between the nutrition styles in terms of willingness to pay a premium price (*p* < 0.05). As a result, *H*_*1*1a_ is accepted, but *H*_10a_ is not accepted.

**TABLE 9:** ANOVA results according to participants' nutrition styles.

Subdimension		Sum of squares	df	Mean square	*F*	Sig.
Overall liking						
	Between groups Within groups Total	0.717 36,875 37,592	2 117 119	0.358 0.315	1137	0.324
Willingness to pay premium price						
	Between groups Within groups Total	6779 65,844 72,623	2 117 119	3390 0.563	6023	0.003*

*Note: p* < 0.05*.

Participants' willingness to pay a premium price was significantly different between dietary style groups (*p* < 0.05). This difference is explained by the Tukey test results in Table 10.

**TABLE 10:** Tukey HSD analysis results regarding participants' dietary style and willingness to pay premium price.

(I) Nutrition style	(J) Nutrition style	Mean difference (I‐J)	95% confidence interval	
			Lower bound	Upper bound
			*s*	*p*
Vegan	Vegetarian Omnivore	0.07500 0.53750*	0.16774 0.16774	0.869 0.005
Vegetarian	Vegan Omnivore	‐,07500 0.46250*	0.16774 0.16774	0.896 0.018
Omnivore	Vegan Vegetarian	‐,53750* ‐,46250*	,16,774 ,16,774	,005 ,018

*Note: p* < 0.05*.

Considering the results of the Tukey HSD analysis regarding participants' willingness to pay a premium price, a significant difference was found in willingness to pay a premium price based on nutrition styles (*p* < 0.05). The analysis results indicate that individuals following a vegan diet demonstrate a higher willingness to pay a premium price compared to both vegetarians and omnivores. Additionally, those following a vegetarian diet also show a higher willingness to pay a premium price than omnivores.

**TABLE 11:** Tukey HSD analysis results of taste, aftertaste impression, and overall liking sensory factors regarding participants' perception of income.

	Perception of income (I)	Perception of income (J)	Mean difference (I‐J)	95% confidence interval	
				Upper bound	Lower bound
				*s*	*p*
Taste	Low	Medium High	0.151 0.336*	0.141 0.118	0.553 0.014
	Medium	Low High	‐,151 0.185	0.141 0.133	0.533 0.346
	High	Low Medium	−0.336* ‐,185	0.118 0.133	0.014 0.346
Aftertaste İmpression	Low	Medium High	0.259 0.333*	0.156 0.131	0.222 0.032
	Medium	Low High	‐,259 0.074	0.156 0.146	0.222 0.869
	High	Low Medium	−0.333* −0,74	0.131 0.146	0.032 0.869
Overall liking	Low	Medium High	,088 0.311*	0.137 0.115	0.797 0.022
	Medium	Low High	−0.088 0.222	0.137 0.129	0.797 0.203
	High	Low Medium	−0.311* −0.222	0.115 0.129	0.022 0.203

*Note: p* < 0.05*.

There is a significant difference (*p* < 0.05 between low and high) according to the income status of the participants concerning the dimensions of taste, aftertaste impression, and overall liking. Firstly, participants with a low‐income perception evaluated the taste attribute with a higher mean compared to those with a high‐income perception (MD = 0.336, *p* = 0.014). A similar difference was observed in the aftertaste impression, where low‐income participants rated the aftertaste more positively compared to high‐income participants (MD = 0.333, *p* = 0.032). Finally, in terms of overall liking, low‐income participants showed a greater preference for the product compared to high‐income participants, with a mean difference of (MD = 0.311, *p* = 0.022). These findings suggest that low‐income participants provided more favorable evaluations of the product's taste, aftertaste, and overall liking compared to high‐income participants. It was determined that those with a low‐income perception found the cookie more delicious and liked the cookie more on the basis of overall liking compared to those with a high‐income perception (Table 11).

## 6 Discussion

As a result of the demographic data, it was concluded that although the participants had different nutrition styles, their education levels and income perceptions were high and they could meet the opportunities required by their nutrition preferences. The overall average of the answers given by the 120 participants in the study for the vegan acibadem cookie during the sensory analysis phase was 4 and above, and the sensory feature was described as “good. When the sensory analysis results were analyzed, all of the nutrition styles gave a score of 4 and above to the overall taste of the developed product. Therefore, there is no significant difference between nutrition styles. On the other hand, this result also indicates that the developed vegan acibadem cookie appeals to all nutrition styles. This result shows that it is possible to create a product that will be liked and requested to be purchased by all dietary styles by meeting the sensory attributes of the product at a certain level. While the correlation of surface appearance and hardness ratio with overall liking was found to be insignificant, other sensory characteristics of the cookie were found to be significant. Hong et al. (2014) conducted a study to understand the relationship between familiarity and cross‐cultural acceptance of an ethnic sweet (Yackwa; a Korean traditional cookie) by Japanese, Korean, and French consumers. It was concluded that the overall liking for the product among French consumers is more closely related to flavor liking than texture liking. This data coincides with one of the results of our study, which is that hardness ratio does not have a significant relationship with overall liking. It is comprehensible that the hardness ratio does not affect the overall liking due to the lack of a comparable product. The classic nonvegan acibadem cookie differs from standard cookies with its unique texture on the inside as well as the crunchiness on the outside. The fact that there is no criterion for the hardness of the cookie for the participants who are experiencing the vegan version for the first time may be one of the factors in the emergence of this result. It seems more possible to conclude that surface appearance is not an important sensory feature for the participants.

Alcântara Brandão et al. (2019) assessed the physicochemical/microbiological properties, nutritional value, and sensory acceptance of cookies formulated with chia. The sample FS30 presented greater acceptance for sensory traits. Also, the sample FS30 showed the highest positive purchase intention (44%), which supports the results of the acceptance test. This conclusion supports the data in this study showing that overall liking has a positive effect on purchase intention. Another notable finding of the study is that there is a significant difference in willingness to pay a premium price among dietary styles. People with a vegan diet have a higher willingness to pay than vegetarians and respondents with omnivore diets. It was also found that vegetarians had a higher willingness to pay than respondents with omnivore diets. This finding is comparable to Shen and Chen (2020) who found that vegetarians were willing to pay more for plant‐based hamburgers than nonvegetarians. Income can influence people's sensory attributes towards a product. For example, children from high‐income families have a preference for chocolate‐based foods, but children from low‐income families are not so keen on these products (Sharif et al. 2017). According to the results of the analysis, a significant difference was found between the groups with respect to income perception in terms of taste, aftertaste impression, and overall liking sensory characteristics. It was determined that those with low‐income perception found the cookie more delicious and liked the cookie more on the basis of overall liking compared to those with good income perception.

Recent literature indicates that individual diet and taste sensitivity may have a correlation, with evidence emphasizing that specific diets can affect taste sensitivities. Mozhdehi et al. (2021) found that the vegetarian or vegan group demonstrated a reduced sensitivity to sweetness and the vegetarian group had a lower perception threshold for bitterness (i.e., caffeine) than the omnivore and vegan groups. Although taste perception varied by diet type, the product scored high in terms of overall liking in all dietary groups in this study. Also, in this study, no significant difference was found between dietary styles and overall liking. The factor that contributes to this can be shown to be the appreciation of the product in all dietary styles. Although this result is consistent with the sensory analysis and significant difference tests, it is not comparable with previous studies. Finally, it can be said that at the point where the overall liking of a product is provided, the difference in taste perception arising from dietary styles is neutralized. On the other hand, it was concluded that the willingness to pay of vegan diet followers was higher than that of vegetarians and omnivores, while the willingness to pay of vegetarians was higher than that of omnivores. It could be inferred that an increase in the product‐based opportunities provided by diets leads to a decrease in the willingness to pay more for a product. There are many more comparable options on the market for an omnivore.

It was found that income perception did not make any significant difference in the willingness to pay more, while there was a difference in taste, aftertaste impression, and overall liking variables. It can be demonstrated that one of the most important factors why those with worse income status find the product more delicious compared to those with better income status is that those with better income perception have a higher level of taste and expect a higher performance in terms of flavor. Delgado et al. (2006) conducted a study to measure Brazilian consumers' perceptions of the toughness of meat sold in grocery stores and examined whether the age, gender, education level, and income of the consumers had any effect on the sensory characteristics of the meat they tasted. As a result of the research, it was concluded that the income of the participants did not have any effect on the sensory characteristics of the meat. This result does not coincide with the significant difference between income perception groups in our study. The overall liking of the acibadem cookie developed within the scope of the study was described as “good” by scoring 4 or more out of 5 points in all dietary styles. It was also found that overall liking positively affected the willingness to pay more. This result is in accordance with Poonnakasem et al. (2016). They examined the effects of different oils on the physicochemical properties, emotion, purchase intent, and consumer liking of sponge cake. They found that overall liking and pleasure are critical traits that affect purchase intention. When both results are analyzed, the high sensory properties of the product in the case of food products provide a certain level of customer satisfaction and willingness to pay more.

## 7 Conclusion

Within the scope of the study, vegan acibadem cookie was developed as an alternative product to the ever‐growing vegan product market. To ensure the developed cookie is vegan, aquafaba was utilized as a substitute for egg whites. This approach not only maintains the product's vegan properties but also supports the zero‐waste concept by reusing chickpea water that would otherwise be discarded, contributing to sustainable production practices. The sensory analysis of the developed product was carried out and the taste of the consumers was evaluated. As a result of the research, the overall liking of the developed product was evaluated as “good” with a score of 4 and above in all dietary styles. As a consequence of the sensory analyses of the developed vegan acibadem cookie, it was determined that the consumers' intention to purchase the developed product was high. In terms of dietary styles, the group with the highest purchase intention was vegans, followed by vegetarians. In accordance with income perception, it was found that those with low‐income perception liked the product more than those with high‐income perception.

## Ethics Statement

Ethical approval for this study was provided by the University of Necmettin Erbakan Social and Humanitarian Sciences Scientific Research Ethics (09/06/23). All participants provided informed consent prior to taking part in this study.

## Conflicts of Interest

The authors declare no conflicts of interest.

## Enlightenment

*This study was conducted by the authors as a master's thesis at Necmettin Erbakan University in 2023 and has been revised for publication as an article in this journal.

## Data Availability

The data of the study are available.

## References

[fsn370141-bib-0001] Alcântara Brandão, N. , B. de Lima , M. Dutra , A. L. Andrade Gaspardi , and M. R. Segura Campos . 2019. “Chia ( *Salvia hispanica* L.) Cookies: Physicochemical/Microbiological Attributes, Nutrimental Value and Sensory Analysis.” Journal of Food Measurement and Characterization 13: 1100–1110. 10.1007/s11694-018-00025-z.

[fsn370141-bib-0002] Allison, P. D. 1990. “Change Scores as Dependent Variables in Regression Analysis.” Sociological Methodology 20: 93–114. 10.2307/271083.

[fsn370141-bib-0003] Al‐Mohaithef, M. 2022. “Prevalence of Vegan/Vegetarian Diet and Eating Behavior Among Saudi Adults and Its Correlation With Body Mass Index: A Cross‐Sectional Study. Frontiers.” Nutrition 9: 966629. 10.3389/fnut.2022.966629. PMC9520056 36185674

[fsn370141-bib-0004] Andersen, B. V. , P. B. Brockhoff , and G. Hyldig . 2019. “The Importance of Liking of Appearance, ‐Odour, ‐Taste and ‐Texture in the Evaluation of Overall Liking. A Comparison With the Evaluation of Sensory Satisfaction.” Food Quality and Preference 71: 228–232. 10.1016/j.foodqual.2018.07.00.

[fsn370141-bib-0005] Anetoh, J. C. , J. O. Nnabuko , V. O. Okolo , and V. C. Anetoh . 2020. “Sensory Attributes of Malt Drinks and Consumer Purchase Decisions.” Journal of Food Products Marketing 26, no. 5: 317–343. 10.1080/10454446.2020.1767748.

[fsn370141-bib-0006] Bezerra, V. S. , O. Freitas‐Silva , L. F. Damasceno , A. M. G. N. Mamede , and L. M. Cabral . 2017. “Sensory Analysis and Consumers Studies of Acai Beverage After Thermal, Chlorine and Ozone Treatments of the Fruits.” Journal of Food Processing and Preservation 41, no. 3: 12961. 10.1111/jfpp.12961.

[fsn370141-bib-0008] Bryła, P. 2018. “Organic Food Online Shopping in Poland.” British Food Journal 120, no. 5: 1015–1027. 10.1108/BFJ-09-2017-0517.

[fsn370141-bib-0010] Chowdhury, S. , A. Meero , A. A. A. Rahman , K. A. Islam , N. M. Zayed , and K. R. Hasan . 2021. “An Empirical Study on the Factors Affecting Organic Food Purchasing Behavior in Bangladesh: Analyzing a Few Factors.” Academy of Strategic Management Journal 20, no. 4: 1–12.

[fsn370141-bib-0011] Clark, M. A. , M. Springmann , J. Hill , and D. Tilman . 2019. “Multiple Health and Environmental Impacts of Foods.” Proceedings of the National Academy of Sciences 116, no. 46: 23357–23362. 10.1073/pnas.1906908116. PMC6859310 31659030

[fsn370141-bib-0012] Conti‐Silva, A. C. , and P. K. de Souza‐Borges . 2018. “Sensory Characteristics, Brand and Probiotic Claim on the Overall Liking of Commercial Probiotic Fermented Milks: Which One Is More Relevant?” Food Research International 116: 184–189. 10.1016/j.foodres.2018.08.011. 30716935

[fsn370141-bib-0014] de Carlo, L. T. 1997. “On the Meaning and Use of Kurtosis.” Psychological Methods 2: 292–307.

[fsn370141-bib-0015] Delgado, E. F. , A. P. Aguiar , E. M. M. Ortega , M. H. F. Spoto , and C. J. C. Castillo . 2006. “Brazilian Consumers' Perception of Tenderness of Beef Steaks Classified by Shear Force and Taste.” Scientia Agricola 63: 232–239.

[fsn370141-bib-0016] Dovi, K. A. , C. Chiremba , J. R. Taylor , and H. L. de Kock . 2018. “Rapid Sensory Profiling and Hedonic Rating of Whole Grain Sorghum‐Cowpea Composite Biscuits by Low‐Income Consumers.” Journal of the Science of Food and Agriculture 98, no. 3: 905–913. 10.1002/jsfa.8536. 28692754

[fsn370141-bib-0017] Drewnowski, A. , and P. Monsivais . 2020. “Taste, Cost, Convenience, and Food Choices.” In Present Knowledge in Nutrition, edited by B. P. M. D. F. Birt , V. A. Stalling , and A. A. Yates , 185–200. Elsevier.

[fsn370141-bib-0018] Dwivedi, A. , T. Nayeem , and F. Murshed . 2018. “Brand Experience and Consumers' Willingness‐To‐Pay (WTP) a Price Premium: Mediating Role of Brand Credibility and Perceived Uniqueness.” Journal of Retailing and Consumer Services 44: 100–107. 10.1016/j.jretconser.2018.06.009.

[fsn370141-bib-0019] Eftimov, T. , G. Popovski , M. Petković , B. K. Seljak , and D. Kocev . 2020. “COVID‐19 Pandemic Changes the Food Consumption Patterns.” Trends in Food Science and Technology 104: 268–272. 10.1016/j.tifs.2020.08.017. 32905099 PMC7462788

[fsn370141-bib-0020] Erem, E. , N. C. Icyer , N. B. Tatlisu , M. Kilicli , G. H. Kaderoglu , and Ö. S. Toker . 2023. “A New Trend Among Plant‐Based Food Ingredients in Food Processing Technology: Aquafaba.” Critical Reviews in Food Science and Nutrition 63, no. 20: 4467–4484. 10.1080/10408398.2021.2002259. 34761963

[fsn370141-bib-0021] Fornell, C. , R. T. Rust , and M. G. Dekimpe . 2010. “The Effect of Customer Satisfaction on Consumer Spending Growth.” Journal of Marketing Research 47, no. 1: 28–35. 10.1509/jmkr.47.1.28.

[fsn370141-bib-0022] French, S. A. , C. C. Tangney , M. M. Crane , Y. Wang , and B. M. Appelhans . 2019. “Nutrition Quality of Food Purchases Varies by Household Income: The SHoPPER Study.” BMC Public Health 19: 1–7. 10.1186/s12889-019-6546-2. 30808311 PMC6390355

[fsn370141-bib-0023] Gheihman, N. 2021. “Veganism as a Lifestyle Movement.” Sociology Compass 15, no. 5: 12877. 10.1111/soc4.12877.

[fsn370141-bib-0024] Ginsberg, C. 2017. “The Market for Vegetarian Foods, the Vegetarian Resource Group.” https://www.vrg.org/nutshell/market.htm. Accecced June 15, 2022.

[fsn370141-bib-0025] Grasso, S. , A. Rondoni , R. Bari , R. Smith , and N. Mansilla . 2022. “Effect of Information on Consumers' Sensory Evaluation of Beef, Plant‐Based and Hybrid Beef Burgers.” Food Quality and Preference 96: 104417. 10.1016/j.foodqual.2021.104417.

[fsn370141-bib-0026] Hajjar, S. T. 2018. “Statistical Analysis: Internal‐Consistency Reliability and Construct Validity.” International Journal of Quantitative and Qualitative Research Methods 6, no. 1: 27–38.

[fsn370141-bib-0027] Hong, J. H. , H. S. Park , S. J. Chung , et al. 2014. “Effect of Familiarity on a Cross‐Cultural Acceptance of a Sweet Ethnic Food: A Case Study With Korean Traditional Cookie (Yackwa).” Journal of Sensory Studies 29, no. 2: 110–125. 10.1111/joss.12087.

[fsn370141-bib-0028] Jalil Mozhdehi, F. , S. Abeywickrema , P. J. Bremer , and M. Peng . 2021. “Comparing Taste Detection Thresholds Across Individuals Following Vegan, Vegetarian, or Omnivore Diets.” Food 10, no. 11: 2704. 10.3390/foods10112704. PMC8619387 34828985

[fsn370141-bib-0029] Joya, K. , N. N. Ramli , M. N. Shamsudin , and N. H. Kamarulzaman . 2022. “Consumers' Willingness to Pay for Food Safety Attributes of Tomato.” British Food Journal 124, no. 3: 701–717. 10.1108/BFJ-02-2021-0164.

[fsn370141-bib-0030] Kumari, S. , and G. V. Muralidhara . 2021. “Nestlé Under Fire Over Unhealthy Product Portfolio: Will the Company Emerge Unscathed?” IUP Journal of Business Strategy 18, no. 3: 38–60.

[fsn370141-bib-0031] Leshem, M. , and S. Shaul . 2022. “Vegans, Vegetarians and Omnivores Differ in Nutrient Hedonics, Salt and Sweet Preference and Flavouring.” Physiology and Behavior 255: 113936. 10.1016/j.physbeh.2022.113936. 35931195

[fsn370141-bib-0032] Loh, H. C. , Y. K. Seah , and I. Looi . 2021. “The COVID‐19 Pandemic and Diet Change.” Progress in Microbes and Molecular Biology 4, no. 1: 0000203. 10.36877/pmmb.a0000203.

[fsn370141-bib-0033] Ma, C. , Z. Song , X. Yan , and G. Zhao . 2018. “Accounting for Tastes: Do Low‐Income Populations Have a Higher Preference for Spicy Foods?” Journal of Chinese Sociology 5: 1–16. 10.1186/s40711-018-0089-2.

[fsn370141-bib-0034] Malhotra, N. K. , and S. Dash . 2016. Marketing Research‐An Applied Orientation. Pearson India Education Services Pvt Ltd.

[fsn370141-bib-0035] Martinelli, E. , and F. de Canio . 2022. “Non‐Vegan Consumers Buying Vegan Food: The Moderating Role of Conformity.” British Food Journal 124, no. 1: 14–30. 10.1108/BFJ-01-2021-0023.

[fsn370141-bib-0036] Montouto‐Graña, M. , S. Cabanas‐Arias , S. Porto‐Fojo , M. L. Vázquez‐Odériz , and M. A. Romero‐Rodríguez . 2012. “Sensory Characteristics and Consumer Acceptance and Purchase Intention Toward Fresh‐Cut Potatoes.” Journal of Food Science 77, no. 1: 40–46. 10.1111/j.1750-3841.2011.02453.x. 22260130

[fsn370141-bib-0037] Mudry, J. , and R. J. Phillips . 2023. “Making Hamburgers Healthy: Plant‐Based Meat and the Rhetorical (Re) Constructions of Food Through Science.” Food, Culture & Society 26, no. 1: 193–208. 10.1080/15528014.2021.1992575.

[fsn370141-bib-0038] Mulder, M. , and S. Zomer . 2017. “Dutch Consumers' Willingness to Pay for Broiler Welfare.” Journal of Applied Animal Welfare Science 20, no. 2: 137–154. 10.1080/10888705.2017.1281134. 28166413

[fsn370141-bib-0039] Mustafa, R. , Y. He , Y. Y. Shim , and M. J. Reaney . 2018. “Aquafaba, Wastewater From Chickpea Canning, Functions as an Egg Replacer in Sponge Cake.” International Journal of Food Science and Technology 53, no. 10: 2247–2255. 10.1111/ijfs.13813.

[fsn370141-bib-0040] Netemeyer, R. G. , B. Krishnan , C. Pullig , et al. 2004. “Developing and Validating Measures of Facets of Customer‐Based Brand Equity.” Journal of Business Research 57, no. 2: 209–224. 10.1016/S0148-2963(01)00303-4.

[fsn370141-bib-0041] Nguyen, H. , and W. V. Wismer . 2019. “A Comparison of Sensory Attribute Profiles and Liking Between Regular and Sodium‐Reduced Food Products.” Food Research International 123: 631–641. 10.1016/j.foodres.2019.05.037. 31285012

[fsn370141-bib-0042] Niederle, P. , and M. N. Schubert . 2020. “How Does Veganism Contribute to Shape Sustainable Food Systems? Practices, Meanings and Identities of Vegan Restaurants in Porto Alegre, Brazil.” Journal of Rural Studies 78: 304–313. 10.1016/j.jrurstud.2020.06.021.

[fsn370141-bib-0043] Noguerol, A. T. , M. J. Pagán , P. García‐Segovia , and P. Varela . 2021. “Green or Clean? Perception of Clean Label Plant‐Based Products by Omnivorous, Vegan, Vegetarian and Flexitarian Consumers.” Food Research International 149: 110652. 10.1016/j.foodres.2021.110652. 34600654

[fsn370141-bib-0044] Patton, M. Q. 2005. Qualitative Research. John Wiley and Sons, Ltd.

[fsn370141-bib-0045] Peter, J. P. 1979. “Reliability: A Review of Psychometric Basics and Recent Marketing Practices.” Journal of Marketing Research 16, no. 1: 6–17. 10.1177/002224377901600102.

[fsn370141-bib-0046] Ploll, U. , and T. Stern . 2020. “From Diet to Behaviour: Exploring Environmental‐ and Animal‐Conscious Behaviour Among Austrian Vegetarians and Vegans.” British Food Journal 122, no. 11: 3249–3265. 10.1108/BFJ-06-2019-0418.

[fsn370141-bib-0047] Poonnakasem, N. , K. D. Pujols , S. Chaiwanichsiri , K. Laohasongkram , and W. Prinyawiwatkul . 2016. “Different Oils and Health Benefit Statements Affect Physicochemical Properties, Consumer Liking, Emotion, and Purchase Intent: A Case of Sponge Cake.” Journal of Food Science 81, no. 1: 165–173. 10.1111/1750-3841.13186. 26661685

[fsn370141-bib-0048] Raikos, V. , H. Hayes , and H. Ni . 2020. “Aquafaba From Commercially Canned Chickpeas as Potential Egg Replacer for the Development of Vegan Mayonnaise: Recipe Optimisation and Storage Stability.” International Journal of Food Science and Technology 55, no. 5: 1935–1942. 10.1111/ijfs.14427.

[fsn370141-bib-0049] Ribeiro, J. C. , R. C. Lima , M. R. Maia , et al. 2019. “Impact of Defatting Freeze‐Dried Edible Crickets (Acheta Domesticus and *Gryllodes sigillatus* ) on the Nutritive Value, Overall Liking and Sensory Profile of Cereal Bars.” LWT 113: 108335. 10.1016/j.lwt.2019.108335.

[fsn370141-bib-0050] Rondoni, A. , E. Millan , and D. Asioli . 2021. “Consumers' Preferences for Intrinsic and Extrinsic Product Attributes of Plant‐Based Eggs: An Exploratory Study in the United Kingdom and Italy.” British Food Journal 123, no. 11: 3704–3725. 10.1108/BFJ-11-2020-1054.

[fsn370141-bib-0051] Ruini, L. F. , R. Ciati , C. A. Pratesi , M. Marino , L. Principato , and E. Vannuzzi . 2015. “Working Toward Healthy and Sustainable Diets: The “Double Pyramid Model” Developed by the Barilla Center for Food and Nutrition to Raise Awareness About the Environmental and Nutritional Impact of Foods.” Frontiers in Nutrition 2, no. 9: 1–6. 25988137 10.3389/fnut.2015.00009 PMC4428432

[fsn370141-bib-0052] Saari, U. A. , C. Herstatt , R. Tiwari , O. Dedehayir , and S. J. Mäkinen . 2021. “The Vegan Trend and the Microfoundations of Institutional Change: A Commentary on Food Producers' Sustainable Innovation Journeys in Europe.” Trends in Food Science and Technology 107: 161–167. 10.1016/j.tifs.2020.10.003.

[fsn370141-bib-0053] Seferoğlu, B. 2012. Evaluation of Bread, Cake and Biscuit Recipes Prepared for Celiac Patients With Chestnut and Gluten Free Flours by Sensory Evaluation Methods (Master's Thesis). Hacettepe University.

[fsn370141-bib-0054] Sharif, M. K. , M. S. Butt , H. R. Sharif , and M. Nasir . 2017. “Sensory Evaluation and Consumer Acceptability.” Handbook of Food Science and Technology 10: 362–386.

[fsn370141-bib-0055] Shen, Y. , and H. Chen . 2020. “Exploring Consumers' Purchase Intention of an Innovation of the Agri‐Food Industry: A Case of Artificial Meat.” Food 9, no. 6: 745. 10.3390/foods9060745. PMC7353571 32512792

[fsn370141-bib-0056] Shim, Y. Y. , R. Mustafa , J. Shen , K. Ratanapariyanuch , and M. J. T. Reaney . 2018. “Composition and Properties of Aquafaba Water Recovered From Commercially Canned Chickpeas.” Journal of Visualized Experiments 132: 56305. 10.3791/56305. PMC5912395 29553544

[fsn370141-bib-0057] Siew, S. W. , M. S. Minor , and R. Felix . 2018. “The Influence of Perceived Strength of Brand Origin on Willingness to Pay More for Luxury Goods.” Journal of Brand Management 25: 591–605. 10.1057/s41262-018-0114-4.

[fsn370141-bib-0058] Singh, A. , and P. Verma . 2017. “Factors Influencing Indian Consumers' Actual Buying Behaviour Towards Organic Food Products.” Journal of Cleaner Production 167: 473–483. 10.1016/j.jclepro.2017.08.106.

[fsn370141-bib-0059] Slabá, M. 2019. “The Impact of Age on the Customers Buying Behaviour and Attitude to Price.” Littera Scripta 12, no. 2: 146–160. 10.36708/Littera_Scripta2019/2/0.

[fsn370141-bib-0060] Śmiglak‐Krajewska, M. , J. Wojciechowska‐Solis , and D. Viti . 2020. “Consumers' Purchasing Intentions on the Legume Market as Evidence of Sustainable Behaviour.” Agriculture 10, no. 10: 424. 10.3390/agriculture10100424.

[fsn370141-bib-0062] Statista . 2023. “Share of Vegans in Select Countries Worldwide in 2022.” https://www.statista.com/statistics/1280066/global‐country‐ranking‐vegan‐share/.

[fsn370141-bib-0063] Vestergren, S. , and M. S. Uysal . 2022. “Beyond the Choice of What You Put in Your Mouth: A Systematic Mapping Review of Veganism and Vegan Identity.” Frontiers in Psychology 13: 848434. 10.3389/fpsyg.2022.848434. 35756214 PMC9231820

[fsn370141-bib-0064] Wirnitzer, K. , M. Motevalli , D. Tanous , et al. 2022. “Who Is Running in the DA‐CH Countries? An Epidemiological Approach of 2455 Omnivorous, Vegetarian, and Vegan Recreational Runners—Results From the NURMI Study (Step 1).” Nutrients 14, no. 3: 677. 10.3390/nu14030677. 35277039 PMC8839653

[fsn370141-bib-0065] Yilmaz‐Ersan, L. , T. Ozcan , and A. Akpinar‐Bayizit . 2020. “Assessment of Socio‐Demographic Factors, Health Status and the Knowledge on Probiotic Dairy Products.” Food Science and Human Wellness 9, no. 3: 272–279. 10.1016/j.fshw.2020.05.004.

